# A128 GLOBAL TRENDS IN TRAINING AND CREDENTIALING GUIDELINES FOR GASTROINTESTINAL (GI) ENDOSCOPY: A SYSTEMATIC REVIEW

**DOI:** 10.1093/jcag/gwac036.128

**Published:** 2023-03-07

**Authors:** N Sabrie, S Seleq, H Homsi, R Khan, N Gimpaya, R Bansal, M Scaffidi, D Lightfoot, S Grover

**Affiliations:** 1 University of Toronto; 2 Gastroenterology; 3 St. Michael's Hospital; 4 Gastroenterology, University of Toronto, Toronto, Canada

## Abstract

**Background:**

Credentialing in GI endoscopy is not a universally standardized process. National guidelines may provide a framework for local training, however in certain settings, training committees set minimal competency requirements that must be met before a clinician can be accredited to practice independently. There is a paucity of literature assessing the inter-societal and geographic variability in guidelines and training requirements in endoscopy.

**Purpose:**

To systematically review the available credentialing guidelines proposed by different GI endoscopy societies and affiliated training committees internationally.

**Method:**

We conducted a systematic review according to the PRISMA guidelines. A comprehensive literature search was performed for credentialing guidelines for GI endoscopy from inception until January 2022. Two reviewers screened and one reviewer abstracted data using a pre-defined data collection form.

**Result(s):**

From the 653 records obtained from our search, 20 credentialing guidelines from 12 different GI societies were ultimately included in the review. These guidelines encompassed the following procedures and outlined the following key-performance indicators; a) Colonoscopy: the recommended minimum number of procedures performed ranged from 150-275 with a minimum cecal intubation and adenoma detection rate of 85-90% and 20-30% respectively; b) EGD: the minimum number of procedures prior to credentialing ranged from 130-1000, the minimum duodenal intubation rate ranged from 95-100%, and the range for minimum number of upper GI bleeds managed was 20-45 (in addition to other procedural KPIs); c) ERCP: the recommended minimum number of procedures prior to credentialing ranged from 100-300 cases with a minimum selective duct cannulation rate of 80-90%. Guidelines for flexible sigmoidoscopy, EUS and capsule endoscopy were also obtained.

**Image:**

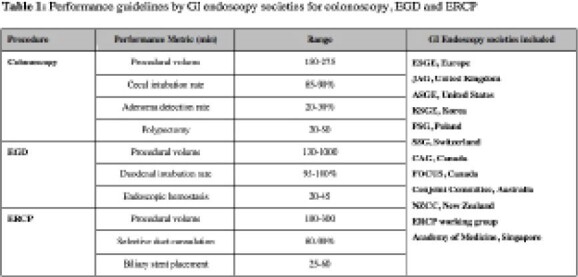

**Conclusion(s):**

There is a general concordance amongst the various international GI societies with regards to minimum procedural volume and performance in key procedural tasks prior to credentialing, however the use of validated education assessment tools was lacking in the majority of guidelines. Additional KPI’s need to be explored for less routinely performed procedures such as EUS and capsule endoscopy.

**Please acknowledge all funding agencies by checking the applicable boxes below:**

None

**Disclosure of Interest:**

None Declared

